# Symptom Patterns, Recovery, and Impact of Long COVID: Findings From a Longitudinal Survey

**DOI:** 10.1093/ofid/ofag040

**Published:** 2026-02-27

**Authors:** Nida Ziauddeen, Marija Pantelic, Margaret E O’Hara, Claire Hastie, Nisreen A Alwan

**Affiliations:** School of Primary Care, Population Sciences and Medical Education, Faculty of Medicine, University of Southampton, Southampton, UK; NIHR Southampton Biomedical Research Centre, University of Southampton and University Hospital Southampton NHS Foundation Trust, Southampton, UK; Brighton and Sussex Medical School, University of Sussex, Falmer, UK; Department of Social Policy and Intervention, University of Oxford, Oxford, UK; Long COVID Support, London, UK; Long COVID Support, London, UK; School of Primary Care, Population Sciences and Medical Education, Faculty of Medicine, University of Southampton, Southampton, UK; NIHR Southampton Biomedical Research Centre, University of Southampton and University Hospital Southampton NHS Foundation Trust, Southampton, UK; NIHR Applied Research Collaboration Wessex, Southampton, UK

## Abstract

**Background:**

Long COVID is a predominantly multisystem, often disabling, condition that develops following SARS-CoV-2 infection. We aimed to characterize the pattern, triggers, and impact of Long COVID symptoms.

**Methods:**

Data from a 1-year follow-up of an online survey originally conducted in November 2020 were used. Surveys were coproduced with people living with Long COVID. Participants were adults with Long COVID following confirmed or probable SARS-CoV-2 infection who were not hospitalized in the first 2 weeks of illness. The baseline survey recruited from social media and online support groups using convenience nonprobability sampling.

**Results:**

Of the 2210 first survey participants invited, 1153 (52%) responded to the follow-up survey. The mean age was 47.7 years (standard deviation 10.6) with 84% females, 83% UK-based, 78% university-qualified, and 90% reporting good to excellent health before SARS-CoV-2 infection. Median duration of illness was 19.8 months (interquartile range, 19.3–20.1) at follow-up. Only 5% of participants reported full recovery, and 45% reported a constant pattern of illness (as opposed to fluctuating or relapsing) compared to 17% at baseline. An equal proportion reported being unable to work at baseline (20.4%) and follow-up (20.6%). However, a higher proportion reported being made redundant or taking early retirement at follow-up (8.9%) than at baseline (2.2%).

**Conclusions:**

This study highlights the prolonged nature of Long COVID as well as the impact on work. This has the potential to widen health inequalities and increase hardship in individuals whose life circumstances and job types may not allow them to make necessary adaptations.

Long COVID occurs following a SARS-CoV-2 infection and is a predominantly multisystem condition that often results in prolonged ill health and functional disability [1, 2]. In March 2024, an estimated 2 million individuals in England and Scotland (3.3% of the population) reported experiencing Long COVID [3]. The majority (71.1%) of people reporting having Long COVID had experienced symptoms for at least 1 year, with 19.2% reporting that it limited their ability to undertake day-to-day activities a lot [3]. The General Practice Patient Survey of people aged 16+ years registered with a General Practice in England randomly sampled annually found that 4.6% in 2024 and 4.0% in 2025 described themselves as having Long COVID, with a further 10% unsure whether they have Long COVID [4]. In the United States, 6.9% of adults reported ever having Long COVID (presence of symptoms for at least 3 months after COVID-19) and 3.4% as currently having Long COVID from the 2022 National Health Interview Survey [5].

A systematic review of 194 studies found that 45% experienced at least 1 unresolved symptom at approximately 4 months after COVID-19 infection regardless of hospitalization status [6]. Factors that have been found to be associated with higher likelihood of developing Long COVID include female sex, older age, higher body mass index, smoking, and preexisting health conditions [7, 8]. The risk of developing Long COVID is higher in those requiring hospitalization or intensive care during the acute phase [7, 9], but the majority of cases are in people with acute infections initially classified as mild due to the higher prevalence of these cases [10].

Few studies have evaluated recovery from prolonged illness particularly in people who were not hospitalized following SARS-CoV-2 infection (during the acute infection stage). A cohort study of 68 patients requiring hospitalization for SARS-CoV-2 infection found that 76.4% reported at least 1 persistent symptom at 12 months with some symptoms (myalgia, cough) decreasing over 12 months, whereas others (sleep disturbance, memory problems) became more frequent [11]. A total of 7.6% of participants with Long COVID in a Spanish cohort study were considered recovered (when all persistent symptoms remitted for at least 3 consecutive months) during follow-up with a median time to recovery of 11.4 months [12].

In November 2020, we collected self-reported data through an online survey using convenience nonprobability sampling and analyzed data from 2550 participants with a median duration of illness of 7.6 months. Most participants described a fluctuating (57.7%) or relapsing (17.6%) pattern of illness with physical activity, stress, and sleep disturbance being common factors that triggered symptoms. A total of 16.9% reported being unable to work and 37% reported loss of income due to COVID-19 illness [13]. The survey was conducted before the development of clinical case definitions for Long COVID; however, our inclusion criteria are broadly consistent with the World Health Organization (WHO) definition: symptoms after confirmed or suspected SARS-CoV-2 infection and affecting daily life [14]. We used symptoms persisting for a minimum of 4 weeks to include in the analysis in line with evidence at the time; however, our analysis found that most participants were ill for at least 2 months (96%) at baseline survey in line with the WHO case definition.

In this study, we aimed to characterize ongoing symptom patterns, triggers, impact of Long COVID on work, and recovery from Long COVID at a 1-year follow-up of our online survey.

## METHODS

Data from a 1-year follow-up of an online Long COVID survey were used [13]. Survey methods have been reported in detail previously [13, 15]. Briefly, the baseline survey was administered in November 2020 (n = 2550). We used convenience nonprobability sampling via social media to ensure recruitment of a community sample of people who identify as living with Long COVID [13]. The survey was restricted to adults aged 18 years or older with confirmed or suspected COVID-19 and who were not hospitalized for the treatment of COVID-19 in the first 2 weeks of experiencing symptoms. Responses were anonymous, but participants who were willing to be contacted for a follow-up survey were asked to consent to future contact and provide contact details. A total of 2210 (86.7%) individuals consented to future contact and provided valid contact details and were invited to complete the follow-up survey in November 2021 (1 year from the baseline survey). Of the 340 individuals not invited, a small proportion (3%) resulted from invalid contact details. No participants withdrew consent between the baseline and follow-up survey. Participants were asked to provide the email address where they received the follow-up invitation email so we could link baseline and follow-up responses. The follow-up survey was only open to participants who took part in the baseline survey. We previously reported on the prevalence of stigma using these follow-up survey data [15].

Participants provided written informed consent (digitally on survey platforms separately before accessing the baseline and follow-up survey). Ethical approval was granted by the University of Southampton Faculty of Medicine Ethics Committee (ID 61434).

### Co-design

The survey was coproduced working with public contributors (M.E.O., C.H.), who have lived the experience of Long COVID and provide peer support to others with Long COVID [16]. N.A.A. also had lived the experience of Long COVID. Public contributor members of the Long COVID Support's COVID-19 Research Involvement Group on Facebook provided feedback on early versions of the questionnaire which was amended accordingly. Qualtrics was used as the platform for the follow-up following feedback from the baseline survey about user-friendliness.

### Measures

Demographic information, baseline health, functional status at start of illness, and preexisting health conditions were captured in the baseline survey. Questions at follow-up included ability to work, current employment status, pattern of illness and impact on health, symptoms that have remained over the longer-term course (symptoms experienced at follow-up), clinical diagnosis of Long COVID and other conditions, and an 8-item patient health questionnaire. Further details on the questions asked to capture the measures listed at follow-up are provided in Supplementary Box 1.

*Current employment status* was captured using a multiple answer question with detailed options including employed (full-time, part-time, phased return to work, working reduced hours), self-employed (with or without employees), unemployed, volunteering, apprenticeship, student, not looking for work, unable to work, retired, made redundant/took early retirement, and other (with an open-text box to provide details). These responses were used to derive mutually exclusive categories: employed/self-employed (full- and part-time), unable to work, unable to work but employed/self-employed, student/volunteer/at home not looking for work, unemployed and looking for work, and retired/other.

*Pattern of illness* was captured through several questions. The first question asked about the nature of symptoms with options for constant (experienced at least 1 symptom every day), fluctuating (but symptoms never completely go away compared to pre-COVID health), relapsing and remitting (have symptom-free periods between relapses), constant for 2 weeks at the start of illness but fluctuating since, constant for 2 weeks at the start of illness but relapsing since, constant for 4 weeks at the start of illness but fluctuating since and constant for 4 weeks at the start of illness but fluctuating since, and other (with an open-text box to provide details). Based on input from the public contributors, we did not define number of days for a symptom-free interval for participants reporting relapsing nature of symptoms because of large variation in the patterns experienced by individuals with Long COVID. For participants reporting relapsing or fluctuating nature of symptoms, additional questions captured detail on length of remission or less intense symptoms, pattern of symptoms (triggered by an identifiable factor, set/cyclical pattern with no identifiable trigger, generally set pattern but occasionally triggered by something), and triggers if known. We asked all participants if symptoms have evolved using a multiple-answer question with response options of stayed the same, intensity has reduced, intensity has increased, hard to estimate, and new symptoms have appeared.

*Symptoms* experienced at follow-up was captured using a list of symptoms developed as part of the co-design of the baseline survey. Common “other” responses to the baseline survey were added as options as well as additional symptoms identified through published research [17] or advocacy/support work by the co-authors.

*Clinical diagnosis of Long COVID* was captured through a multiple-answer question with response options of: yes—have Long COVID as a diagnosis on my health record, not officially diagnosed but doctors suspect I have Long COVID, not been diagnosed with Long COVID and doctors do not suspect I have Long COVID, not sure if officially diagnosed with Long COVID, and tested positive for COVID-19 but not received a clinical diagnosis of Long COVID. Diagnosis of myalgic encephalomyelitis/chronic fatigue syndrome (ME/CFS) diagnosis since COVID-19 infection was captured using a binary (yes/no) question. Any other new diagnoses since COVID-19 infection were also captured using a binary (yes/no) with an open-text box to specify details of new diagnosis. This was because patient and public engagement work indicated that people with Long COVID commonly got a diagnosis of ME/CFS instead of Long COVID or received diagnosis based on some of the symptoms experienced.

*Recovery* was self-defined by the participant and captured using an “Yes, I consider myself fully recovered (feeling as healthy as I did before infection and able to function at the same level of activity)” option to a question on how the participant would currently describe their health. The other response options to this question were: “still experiencing symptoms,” “feel far from recovery,” “feel stable but have lower level of health and activity,” “feel stable and close to baseline health/recovery,” “feel potential for relapse,” and “unsure because symptoms come and go.” Participants who responded unsure were included in all analyses because only a small proportion of participants chose this response alone, with the majority selecting 1 or more of the available response options. Based on responses to other questions in the survey and input from public contributors, we are confident that participants were unsure how to describe their current health but were still experiencing Long COVID. Participants who chose the recovered option were additionally asked “How long they were symptom-free before considering yourself completely recovered” and “Over what period did your Long COVID last.”

### Statistical Analysis

Data were downloaded from Qualtrics after the survey was taken offline. Statistical analysis was carried out using Stata. Complete case analysis was carried out as missing data were minimal.

Descriptive percentages and summary statistics were generated for the full sample and stratified by those with and without Long COVID diagnosis. Univariate comparisons between those with and without Long COVID diagnosis were carried out using *t*-test for continuous variables and chi-squared test for categorical variables.

Questions on employment status, symptom pattern, job loss, and income loss resulting from COVID-19 illness were included in both baseline and follow-up surveys, and variables were derived to characterize the change based on the responses given at each survey. Logistic regression was used to examine the association between having received a Long COVID diagnosis at follow-up and symptom pattern, work status, and post-COVID-19 functional status at baseline [18]. Initial univariable analysis was followed by multivariable models adjusting for age, gender, ethnicity, highest educational attainment, smoking status at baseline survey, health before COVID-19, preexisting health condition, household income (model 1), and the other exposures considered (eg, symptom pattern was adjusted for employment status at baseline and post-COVID-19 functional status score at 6 weeks from start of illness).

## RESULTS

Of the 2210 participants invited, 1153 responded to the follow-up survey in November 2021. The mean age was 47.7 years (standard deviation 10.6) with 84% female, 95% of White ethnicity, 78% with university education, and 83% were based in the United Kingdom (Table 1). There was no difference between descriptives, baseline illness pattern, or work pattern between responders and nonresponders (Supplementary Table 1). Ninety percent reported good to excellent health before SARS-CoV-2 infection and 46.8% reported having a preexisting health condition at the time of SARS-CoV-2 infection. Median duration of Long COVID illness was 19.8 months (interquartile range, 19.3–20.1) at follow-up. Fewer than half (48.6%, n = 530) reported having a clinical diagnosis of Long COVID on their health record and a further 28% reported that doctors suspected Long COVID but did not have an official diagnosis. A total of 9.8% had received a diagnosis of ME/CFS and 41.6% had received a new diagnosis since SARS-CoV-2 infection. Only 5% of participants (n = 54) reported full recovery.

**Table 1.** Demographics and Symptom Patterns in the Full Sample and Stratified by Those With/Without Long COVID Diagnosis

Reporting Timepoint	Variable	Full Sample		Long COVID Diagnosis				*P* Value^a^
				No/Not Sure		Yes		
		n	%	n	%	n	%	
	Total n	1153	…	561	…	530	…	
Baseline	Age, years (mean ± standard deviation)	47.7 ± 10.6	…	48.2 ± 11.1	…	47.0 ± 10.0	…	.055
Baseline	Age, categorized							
	18–30	63	5.5	28	5.0	31	5.9	
	31–45	415	36.0	204	36.4	194	36.7	
	46–59	519	45.1	230	41.0	255	48.2	
	≥60	155	13.5	99	17.6	49	9.3	
Baseline	Gender							
	Male	173	15.0	103	18.4	57	10.8	.001
	Female	965	83.8	448	80.0	469	88.5	
	Other	14	1.2	9	1.6	4	0.8	
Baseline	Ethnicity							
	White	1096	95.4	535	95.4	502	95.1	.17
	Mixed/multiple ethnic groups	23	2.0	7	1.3	15	2.8	
	Asian	24	2.1	15	2.7	9	1.7	
	Black/African/Caribbean	4	0.4	2	0.4	2	0.4	
	Other	2	0.2	2	0.4	–	–	
Baseline and follow-up	Country of residence							
	UK—England	767	67.1	355	63.7	365	69.4	.08
	UK—Scotland	111	9.7	57	10.2	52	9.9	
	UK—Wales	57	5.0	27	4.9	28	5.3	
	UK—Northern Ireland	9	0.8	8	1.4	1	0.2	
	Outside the UK	200	17.5	110	19.8	80	15.2	
	Africa	5	0.4	4	0.7	–	–	
	Australia and New Zealand	5	0.4	3	0.5	2	0.4	
	Europe	98	8.6	39	7.0	37	7.0	
	South/Central America and Caribbean	2	0.2	1	0.2	1	0.2	
	North America	82	7.2	45	8.1	34	6.5	
	Asia	5	0.4	5	0.9	–	–	
Baseline	Baseline health before COVID-19 infection							
	Poor	10	0.9	7	1.3	3	0.6	.27
	Fair	103	8.9	61	10.9	40	7.6	
	Good	297	25.8	141	25.1	139	26.2	
	Very good	478	41.5	226	40.3	223	42.1	
	Excellent	265	23.0	126	22.5	125	23.6	
Baseline and follow-up	Education							
	No formal qualifications	12	1.0	9	1.6	2	0.4	.32
	O levels or equivalent	97	8.4	51	9.1	42	7.9	
	A levels or equivalent	149	13.0	76	13.6	64	12.1	
	University degree or above	892	77.5	423	75.7	420	79.4	
Follow-up	Employment status							
	Employed/self-employed	760	66.0	379	67.7	332	62.6	.053
	Unable to work	237	20.6	109	19.5	121	22.8	
	Made redundant/took early retirement	102	8.9	66	8.9	31	8.9	
	Unable to work but employed/self-employed	59	5.1	15	2.7	42	7.9	
	Student/volunteer/at home not looking for work	77	6.7	45	8.0	28	5.3	
	Unemployed and looking for work	17	1.5	11	2.0	6	1.1	
	Retired/other	2	0.2	1	0.2	1	0.2	
Follow-up	Loss of income due to Long COVID							
	No	613	53.3	346	61.7	228	43.2	<.001
	Yes	538	46.7	215	38.3	300	56.8	
Baseline	Household size							
	1 (lives alone)	219	19.1	112	20.0	95	18.1	.68
	2	393	34.3	186	33.2	193	36.8	
	3	201	17.5	100	17.9	82	15.6	
	4	241	21.0	115	20.5	110	21	
	5 or more	92	8.0	47	8.4	44	8.4	
Baseline	Preexisting condition							
	No	614	53.3	307	54.7	266	50.2	.13
	Yes	539	46.8	254	45.3	264	49.8	
Follow-up	Duration of illness							
	12–<15 mo	64	5.6	38	6.8	24	4.6	.21
	15–<18 mo	49	4.3	27	4.8	21	4.0	
	≥18 mo	1034	90.1	493	88.4	482	91.5	
Follow-up	Time since last Long COVID symptom							
	Never had a symptom-free day	677	59.2	271	48.5	386	73.0	<.001
	<2 wk	259	22.7	156	27.9	88	16.6	
	2–4 wk	42	3.7	25	4.5	12	2.3	
	1–<2 mo	31	2.7	17	3.0	12	2.3	
	2–<3 mo	18	1.6	14	2.5	3	0.6	
	3–<4 mo	20	1.8	16	2.9	3	0.6	
	4–<6 mo	30	2.6	19	3.4	10	1.9	
	≥6 mo	66	5.8	41	7.3	15	2.8	
Follow-up	Diagnosis of Long COVID							
	No	53	4.9	53	9.4	–	–	…
	Not sure	129	11.8	129	23.0	–	–	
	Have test confirmation of initial COVID infection but no/not sure clinical diagnosis of Long COVID	73	6.7	73	13.0	–	–	
	No official diagnosis but doctors suspect I have Long COVID	306	28.0	306	54.5	–	–	
	Yes, Long COVID as diagnosis on health record	530	48.6	–	–	530	100	
Follow-up	Diagnosis of ME/CFS post-COVID-19 infection							
	No	989	90.2	523	93.4	456	86.7	<.001
	Yes	107	9.8	37	6.6	70	13.3	
Follow-up	New diagnosis post-COVID-19 infection							
	No	638	58.4	360	64.5	270	51.4	<.001
	Yes	455	41.6	198	35.5	255	48.6	
Follow-up	Reinfected with COVID-19 since initial infection							
	No	958	86.8	485	86.8	459	86.9	.93
	Yes	146	13.2	74	13.2	69	13.1	
Follow-up	Symptoms over the course of the illness							
	Stayed the same	48	4.3	29	5.2	17	3.2	<.001
	Symptom intensity reduced	450	40.4	258	46.2	178	33.8	
	Symptom intensity increased	23	2.1	12	2.2	11	2.1	
	Hard to estimate intensity	92	8.3	57	10.2	32	6.1	
	New symptoms appeared	202	18.1	86	15.4	111	21.1	
	Symptom intensity decreased and new symptoms appeared	211	18.9	94	16.8	111	21.1	
	Symptom intensity increased and new symptoms appeared	47	4.2	10	1.8	36	6.8	
	Symptom intensity increased for some symptoms, decreased for other symptoms and new symptoms appeared	5	0.4	2	0.4	3	0.6	
Follow-up	Symptom pattern							
	Constant	501	44.8	195	35.7	291	55.4	<.001
	Fluctuating	296	26.5	157	28.8	123	23.4	
	Relapsing/remitting	110	9.8	73	13.4	31	5.9	
	Constant for 2 wk at the start and then fluctuating	19	1.7	10	1.8	9	1.7	
	Constant for 4 wk at the start and then fluctuating	69	6.2	33	6.0	33	6.3	
	Constant for 2 wk at the start and then relapsing	11	1.0	9	1.6	1	0.2	
	Constant for 4 wk at the start and then relapsing	49	4.4	37	6.8	6	1.1	
	Other	…	…	…	…	…	0.0	
	Constant for few months at the start and then fluctuating	13	1.2	4	0.7	9	1.7	
	Constant for few months at the start and then relapsing	19	1.7	12	2.2	7	1.3	
	Varied throughout course of illness	8	0.7	5	0.9	3	0.6	
	Generally constant with some symptom-free periods and improvement over the longer term	21	1.9	11	2.0	10	1.9	
	Getting worse	2	0.2	–	–	2	0.4	
Follow-up	Trigger/pattern of symptoms for those reporting fluctuating or relapsing nature of illness (n = 637)							
	Usually triggered by/flares up due to an identifiable factor	256	40.2	142	38.1	108	43.6	.27
	Set/cyclical pattern with no identifiable trigger	99	15.5	60	16.1	36	14.5	
	Generally follows a set/cyclical pattern but occasionally triggered by/flares up due to something	192	30.1	116	31.1	71	28.6	
	Appears random and unable to identify pattern or trigger	62	9.7	42	11.3	19	7.7	
	Usually triggered by/flares up due to an identifiable factor but sometimes no identifiable trigger	28	4.4	13	3.5	14	5.7	
Follow-up	Triggers							
	Physical activity	502	43.5	269	48.0	222	41.9	.04
	Stress	466	40.4	252	44.9	202	38.1	.02
	Work	246	21.3	124	22.1	117	22.1	.99
	Diet	168	14.6	97	17.3	68	12.8	.04
	Hormonal changes	198	17.2	104	18.5	92	17.4	.61
	Cognitive effort	310	26.9	146	26.0	155	29.3	.23
	Social effort	287	24.9	140	25.0	140	26.4	.58
	Emotional effort	264	22.9	120	21.4	137	25.9	.08
	Body posture	131	11.4	69	12.3	59	11.1	.55
	Talking/shouting/singing including voice projection	164	14.2	73	13.0	89	16.8	.08

Abbreviation: ME/CFS, myalgic encephalomyelitis/chronic fatigue syndrome.

^a^Comparisons between those with and without Long COVID diagnosis used *t*-test for continuous variables and chi-squared test for categorical variables.

### Symptom Patterns and Triggers

More than two thirds (68.4%, n = 792) of participants reported still experiencing Long COVID symptoms. Of the 792 participants, 145 only selected the still experiencing symptoms option, 129 also reported feeling far from recovery, and 83 reported experiencing symptoms and feeling stable at a lower level of health and activity than pre-COVID (Supplementary Figure 1). Eighty-four participants (7.6%) reported feeling stable and close to the pre-COVID level of health and activity, 38 participants reported feeling unsure about their health as symptoms come and go and a further 184 participants reported feeling unsure in combination with other response options.

Forty-five percent reported a constant pattern of illness, with the proportion higher in those with a Long COVID diagnosis (55.4%) than those without (35.7%) (Table 1). A higher proportion of participants without a Long COVID diagnosis (13.4%) reported relapsing/remitting symptom pattern than those with a diagnosis (5.9%). Of 637 participants reporting fluctuating or relapsing pattern, 40% reported that their illness (change in symptom intensity or relapse) was usually triggered by an identifiable factor. A further 30% reported that their illness (change in symptom intensity or relapse) generally followed a set/cyclical pattern but was occasionally triggered by something, and 10% reported that they had been unable to identify a trigger. Common triggers for change in symptom intensity or relapse were physical activity (44%), stress (40%), cognitive effort (27%), social effort (25%), and work (21%).

Forty percent of participants reported decrease in symptom intensity over the course of the illness (since infection), with a further 19% also reporting decrease in symptom intensity alongside the appearance of new symptoms. A total of 73% of participants with and 48.5% of those without a Long COVID diagnosis reported never having had a symptom-free day. Most common symptoms at follow-up were exhaustion (67.8%), postexertional symptom exacerbation (62.5%), and cognitive dysfunction (brain fog 62.3%, poor concentration 51.1%, memory problems 49.2%, and difficulty processing information 48.6%) (Supplementary Table 2). Postexertional symptom exacerbation and difficulty processing information were not collected at baseline but exhaustion and cognitive dysfunction were also the most common symptoms at baseline and in line with existing evidence and international case definitions (eg, WHO [1]) for Long COVID.

**Figure 1.**
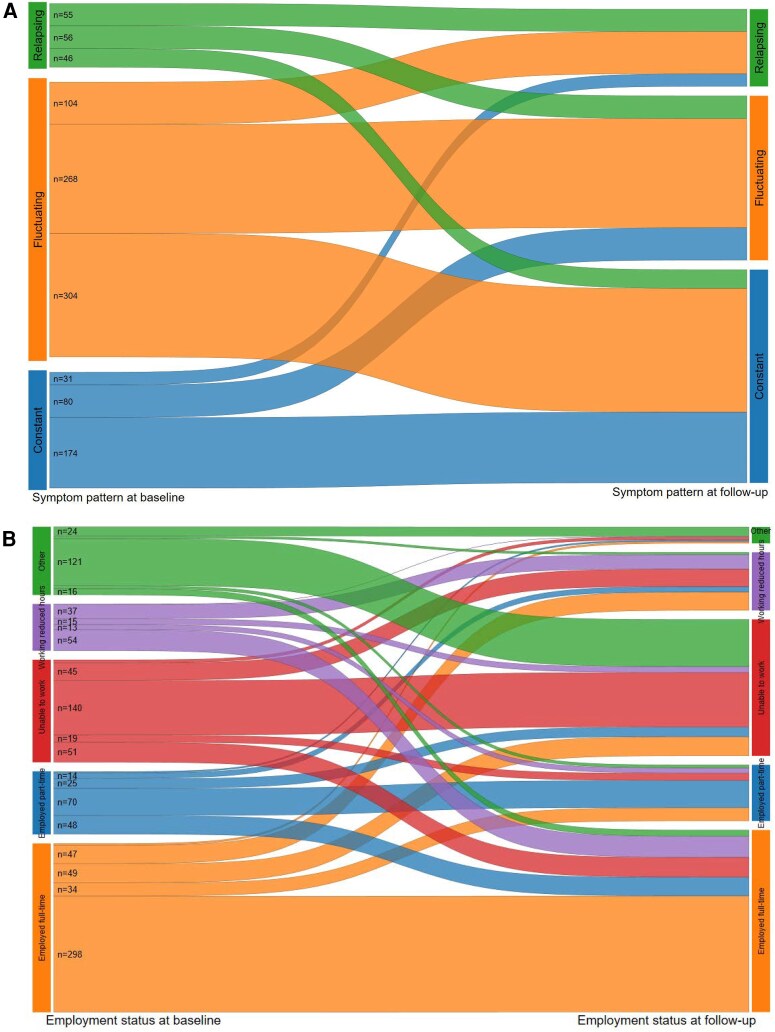

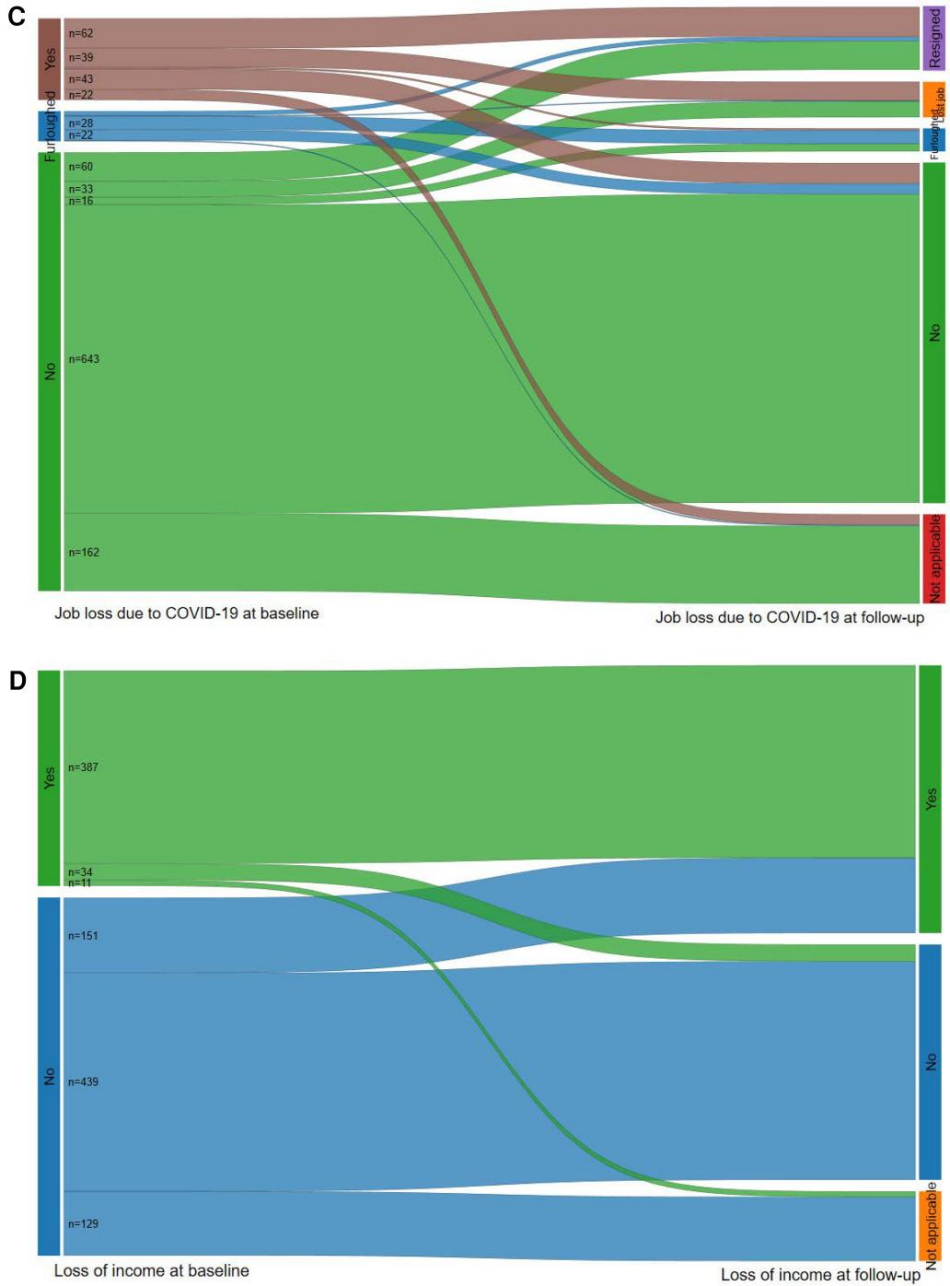
Change in symptom pattern (*A*), employment status (*B*), job loss (*C*), and loss of income (*D*) due to COVID-19 illness between baseline and follow-up. The n has not been presented for transitions with sample size less than 10 but the transitions have been presented.

Among participants reporting experiencing a constant symptom pattern at baseline survey (n = 285), more than half (61.1%) reported continuing to experience a constant pattern of illness, 28.1% experiencing fluctuating, and 10.9% experiencing a relapsing pattern at follow-up survey(Figure 1*A*). Among those reporting experiencing a relapsing symptom pattern at baseline survey (n = 157), 35.0% continued to experience a relapsing pattern, 35.7% experienced fluctuating, and 29.3% experienced a constant symptom pattern at follow-up survey.

Being ill affected leisure activities (79.4%), social activities (72.2%), domestic chores (67.2%), job (61.1%), mental health (59.0%), and personal relationships (51.4%) at the follow-up survey (Table 2). Data on illness affecting personal relationships were not collected in the baseline survey but the activities most commonly affected by illness from the baseline survey were the same as in the follow-up survey. A higher proportion reported illness affecting domestic chores (86.7% at baseline, 67.2% at follow-up) and work (76.1% at baseline, 61.1% at follow-up) at baseline.

**Table 2.** **Changes in Work and Impact of Illness Between Baseline and Follow-Up in the Full Sample and Stratified by Those With/Without Long** COVID **Diagnosis**

Variable	Full Sample				Long COVID Diagnosis							
					No/Not Sure				Yes			
	Baseline		Follow-up		Baseline		Follow-up		Baseline		Follow-up	
	n	%	n	%	n	%	n	%	n	%	n	%
Employment status												
Employed/self-employed	715	62.0	760	66.0	364	64.9	379	67.7	310	58.5	332	62.6
Unable to work	235	20.4	237	20.6	75	13.4	109	19.5	150	28.3	121	22.8
Made redundant/took early retirement	25	2.2	102	8.9	11	2.2	66	8.9	12	2.2	31	8.9
Unable to work but employed/self-employed	–	–	59	5.1	–	–	15	2.7	–	–	42	7.9
Student/volunteer/at home not looking for work	80	6.9	77	6.7	47	8.4	45	8.0	30	5.7	28	5.3
Unemployed and looking for work	19	1.6	17	1.5	14	2.5	11	2.0	4	0.8	6	1.1
Retired/other	104	9.0	2	0.2	61	10.9	1	0.2	36	6.8	1	0.2
Job loss due to COVID-19 illness												
Not applicable	–	–	186	16.1	–	–	109	19.4	–	–	67	12.6
No	914	79.7	710	61.6	449	80.3	343	61.1	416	78.9	325	61.3
No but was furloughed	63	5.5	48	4.2	39	7.0	29	5.2	22	4.2	19	3.6
Yes	170	14.8	209	18.1	71	12.7	80	14.2	89	16.9	119	22.5
Lost job	–	–	74	6.4	–	–	21	3.7	–	–	48	9.1
Resigned from or left job	–	–	135	11.7	–	–	59	10.5	–	–	71	13.4
Had time off sick												
Not applicable	–	–	205	17.8	–	–	116	20.7	–	–	81	15.3
No	307	26.6	122	10.6	195	34.8	86	15.4	93	17.6	24	4.6
Furloughed	45	3.9	33	2.9	29	5.2	19	3.4	14	2.6	12	2.3
Unpaid leave	–	–	80	7.0	–	–	35	6.3	–	–	36	6.8
Yes	801	69.5	634	55.1	337	60.1	264	47.1	423	79.8	341	64.6
Unpaid and sick leave	–	–	46	4.0	–	–	21	3.8	–	–	24	4.6
Furloughed, unpaid leave, and/or sick leave	–	–	6	0.5	–	–	5	0.9	–	–	1	0.2
Furloughed and sick leave	–	–	24	2.1	–	–	14	2.5	–	–	9	1.7
Time off sick in days, categorized (n = 656)												
1 mo or less	250	33.1	184	28.0	141	43.8	114	40.6	92	23.2	59	17.0
>1–3 mo	226	29.9	119	18.1	95	29.5	68	24.2	122	30.7	45	13.0
>3–6 mo	230	30.4	119	18.1	71	22.0	40	14.2	148	37.3	74	21.3
>6–12 mo	50	6.6	121	18.4	15	4.7	38	13.5	35	8.8	79	22.8
>12 mo	–	–	113	17.2	–	–	21	7.5	–	–	90	25.9
Loss of income due to COVID-19 illness												
Not applicable	–	–	140	12.2	–	–	84	15.0	–	–	46	8.7
No	720	62.5	473	41.1	375	66.8	262	46.7	300	56.6	182	34.5
Yes	433	37.6	538	46.7	186	33.2	215	38.3	230	43.4	300	56.8
Being ill affected												
Self-care	563	49.4	383	33.2	236	42.9	148	26.4	306	57.7	225	42.5
Childcare	406	35.6	223	19.3	179	32.6	78	13.9	211	39.8	139	26.2
Caring for other adults	310	27.2	264	22.9	129	23.5	92	16.4	169	31.9	167	31.5
Personal relationships	–	–	593	51.4	–	–	233	41.5	–	–	343	64.7
Domestic chores	988	86.7	775	67.2	446	81.1	319	56.9	497	93.8	428	80.8
Job	868	76.1	705	61.1	372	67.6	273	48.7	458	86.4	410	77.3
Leisure activities	997	87.5	916	79.4	464	84.4	409	72.9	492	92.8	473	89.3
Social activities	890	78.1	832	72.2	384	69.8	342	61.0	464	87.6	460	86.8
Mental health	711	62.4	680	59.0	336	61.1	319	56.9	346	65.3	341	64.3
Daily activities	–	–	471	40.9	–	–	172	30.7	–	–	285	53.8
Other	–	–	97	8.4	–	–	48	8.6	–	–	43	9.1

Participants experiencing a relapsing symptom pattern at baseline were less likely to report having a Long COVID diagnosis at follow-up (adjusted odds ratio [aOR] 0.43; 95% confidence interval [CI], 0.28-0.67) compared to those experiencing a constant pattern of illness (Table 3). Compared to participants experiencing none or negligible functional limitations at 6 weeks from start of illness, participants experiencing moderate (aOR 2.97; 95% CI, 1.75-5.05) and severe (aOR 3.82; 95% CI, 2.23-6.54) functional limitations were more likely to have a Long COVID diagnosis.

**Table 3.** **Association Between Having a Long** COVID **Diagnosis at Follow-Up and Symptom Pattern, Work Status, and Post-**COVID**-19 Functional Status at Baseline**

…	Unadjusted		Model 1		Model 2	
	OR	95% CI	OR	95% CI	OR	95% CI
Symptom pattern at baseline						
Constant	Ref	…	Ref	Ref	Ref	Ref
Fluctuating	1.01	0.76-1.33	0.97	0.72-1.30	0.94	0.69-1.27
Relapsing	0.39	0.25-0.59	0.37	0.24-0.57	0.43	0.28-0.67
Employment status at baseline						
Employed full-time	Ref	…	Ref	…	Ref	…
Employed part-time	1.07	0.73-1.55	1.12	0.76-1.65	1.12	0.75-1.67
Unable to work	2.48	1.79-3.44	2.87	2.02-4.08	2.46	1.72-3.52
Working reduced hours	1.63	1.07-2.48	1.61	1.04-2.50	1.38	0.88-2.16
Not looking for work (student, retired, homemaker)	0.64	0.44-0.94	0.67	0.44-1.02	0.67	0.44-1.04
Post-COVID-19 functional status score						
No/negligible functional limitations	Ref	…	Ref	…	Ref	…
Slight functional limitations	1.56	0.90-2.68	1.40	0.80-2.44	1.43	0.82-2.51
Moderate functional limitations	3.22	1.93-5.36	2.98	1.76-5.04	2.97	1.75-5.05
Severe functional limitations	4.38	2.61-7.36	3.96	2.32-6.76	3.82	2.23-6.54

Model 1: adjusted for age, gender, ethnicity, highest educational attainment, smoking status at baseline survey, health before COVID-19, preexisting health condition, and household income.

Model 2: model 1 plus other exposures considered (eg, symptom pattern model is adjusted for employment status at baseline and post-COVID-19 functional status score at 6 wk from start of illness).

### Impact on Work

An equal proportion reported being unable to work at baseline (20.4%, n = 235) and follow-up (20.6%, n = 237) (Table 2). However, a higher proportion reported being made redundant or taking early retirement at follow-up (8.9%, n = 102) than at baseline (2.2%, n = 25). A further 59 participants (5.1%) reported being employed but unable to work (on paid or unpaid sick leave) at follow-up.

A higher proportion of participants with a Long COVID diagnosis reported being unable to work at follow-up (22.8%), which was a decrease from baseline (28.3%). The pattern was the opposite in participants without a Long COVID diagnosis, with 13.4% reporting being unable to work at baseline increasing to 19.5% at follow-up. A higher proportion of participants with a Long COVID diagnosis reported being employed and unable to work (7.9%) than those without a diagnosis (2.7%). Compared to participants employed full-time at baseline survey, those reporting being unable to work (aOR 2.56; 95% CI, 1.72-3.52) were more likely to report having a Long COVID diagnosis (Table 3). Participants reporting working reduced hours (aOR 1.61; 95% CI, 1.04-2.50) were more likely to report a Long COVID diagnosis, but this was attenuated on adjusting for symptom pattern and functional status.

A total of 209 (18.1%) participants reported losing, resigning from, or leaving their job because of Long COVID at follow-up compared with 170 (14.8%) participants at baseline. A higher proportion of participants with a Long COVID diagnosis reported resigning from or leaving their job (13.4%) than those without a diagnosis (10.5%).

A total of 307 (26.6%) participants reported not taking time off sick due to Long COVID at baseline, which decreased to 122 (10.6%, 4.6% in those with a Long COVID diagnosis and 15.4% in those without) at follow-up. A total of 11.5% reported taking unpaid leave. Of the 656 individuals reporting length of time off sick, 354 (54%) were off sick for more than 3 months, with 113 (17.2%) being off sick for more than 12 months at follow-up. A total of 169 participants with a Long COVID diagnosis reported being off sick for 6 months or more (48.7%), more than double the proportion in those with a diagnosis (21.0%, n = 59). Nearly half (47%, n = 538) reported a loss in income, increasing from 37.6% (n = 433) at baseline.

More than half (53.2%) of participants that reported being unable to work at baseline were still unable to work at follow-up with 17.1% reporting working reduced hours (Figure 1*B*). A total of 26.6% participants who reported being unable to work at baseline reported being employed full- or part-time at follow-up. A high proportion of participants that reported being employed at baseline were employed at follow-up but 11.3% of those employed full-time and 15.5% employed part-time at baseline reported being unable to work at follow-up. A total of 10.9% of participants employed full-time and 8.7% employed part-time at baseline reported working reduced hours at follow-up. Nearly one third (30.8%) of participants who reported working reduced hours or a phased return to work at baseline were still working reduced hours or a phased return at follow-up. A total of 17.5% of participants who were furloughed at baseline reported losing (3.2%) or resigning from or leaving their job (14.3%) at follow-up (Figure 1*C*). Twenty-one percent of participants who reported no loss of income because of COVID-19 illness at baseline reported a loss of income at follow-up (Figure 1*D*).

### Recovery

**Figure 2.**
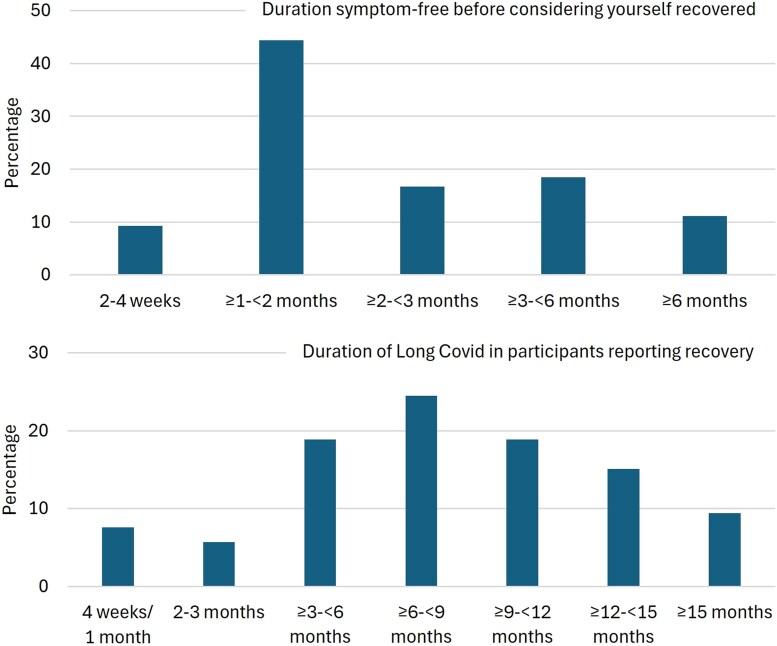
Duration symptom-free and duration of Long COVID before reporting recovery (n = 54).

Of the 54 participants reporting full recovery in this sample, 13.3% (n = 7) reported experiencing Long COVID symptoms for 1–3 months before recovering (Figure 2). Thirteen participants (24.5%) reported experiencing Long COVID for ≥12 months before recovery. Most participants were symptom-free for at least 1–2 months (44.4%, n = 24) before considering themselves recovered, with 6 participants (11.1%) reporting being symptom-free for more than 6 months before considering themselves recovered.

## DISCUSSION

Findings from this longitudinal survey indicate that Long COVID remains a debilitating illness, with only 5% (n = 54) of the study sample reporting recovery. At an average of 20 months from infection, 59% of participants reported never having had a symptom-free day, 59% said it affected their mental health, and 61% said it affected their work. Less than half (48.6%) the participants had an official diagnosis of Long COVID on their medical record.

A higher proportion of those with a Long COVID diagnosis reported being unable to work at follow-up but the proportion decreased from baseline, whereas the proportion unable to work increased from baseline to follow-up in those without a Long COVID diagnosis. We found that 30.8% of participants who reported working reduced hours or a phased return to work at baseline were still working reduced hours or on phased return at follow-up 1 year later. This is in line with findings from a qualitative study in Belgium that found that the fluctuating and cyclical nature of Long COVID could hinder return to work and was not always possible for months after infection [19]. A cross-sectional study in Spain of 77 participants with Long COVID (mean illness duration, 20.7 months) found that 47% were on sick leave (mean duration, 12 months) and 16% had returned to work on reduced hours [20]. Findings from a cross-sectional study of 119 individuals with Long COVID recruited online found that 54.6% had experienced long periods of being unable to work, 34.5% had lost their job, and 7.6% had experienced financial difficulty [21]. Although the proportions are different to those in our study sample (some of which may be due to the different length of follow-up), the pattern is similar, indicating the impact of Long COVID on people's ability to work. People whose life circumstances or job types do not allow them the flexibility to adapt life routines to avoid activities that trigger symptom intensity or relapses may widen health and socioeconomic inequalities.

A total of 41.6% of participants reported receiving a new diagnosis and 9.8% reported receiving a diagnosis of ME/CFS post-COVID-19. This is in line with findings in other studies of chronic long-term conditions including heart disease, diabetes, and ME [22–24]. A study in Australia found that 79% of the 33 included participants with Long COVID met the diagnostic criteria for postural orthostatic tachycardia syndrome [25].

### Limitations and Strengths

This is a nonrepresentative sample recruited through online support groups and generally through social media using convenience nonprobability sampling. This is likely a highly self-selecting group and could overrepresent those who are more severely affected or more engaged in research. The study sample was recruited at a time when research into Long COVID was still in its infancy. Participants were predominantly White, female, and with higher educational attainment; findings therefore cannot be generalized to groups not represented among participants and cannot be used to calculate the prevalence of severity levels among people with Long COVID. The data were collected through online questionnaires, and we attempted to keep both surveys as short as possible to be manageable for participants. There is a possibility of recall bias in the baseline survey as the data about the acute stage were collected retrospectively; however, ongoing symptoms/experiences in both baseline and follow-up surveys were reported at the time point of data collection. Individuals with more symptoms or more severe symptoms may have been more likely to respond to the follow-up survey. The follow-up survey was available to complete for a 4-week period, and a 52% follow-up rate was achieved.

A key strength of this survey is that both baseline and follow-up surveys were co-produced with people with Long COVID. The initial idea for the survey came from people with Long COVID, and they were involved throughout the research. We additionally implemented feedback in an iterative manner from people with Long COVID. They were invited to give feedback from a post in the COVID-19 Research Involvement Group, and group members tested initial versions of both surveys. We changed survey platforms from the first to the second survey so that participants had the option of returning to complete the survey at a later date based on feedback that this made it more feasible for participants to participate in the study making it more inclusive. We captured lived experience with our analysis demonstrating that many people are still struggling to get recognition and diagnosis of Long COVID.

This research demonstrates the continued impact of Long COVID on daily activities and work in a sample of predominantly healthy adults before infection. Further research in a representative population sample is needed to characterize the effect on working patterns in people with Long COVID, particularly in those who may be less able to take time off to recover because of less flexible or more physically demanding occupations, and the effect of clinical recognition of Long COVID and workplace accommodations.

## Supplementary Material

ofag040_Supplementary_Data

## Supplementary Data

Supplementary materials are available at *Open Forum Infectious Diseases* online. Consisting of data provided by the authors to benefit the reader, the posted materials are not copyedited and are the sole responsibility of the authors, so questions or comments should be addressed to the corresponding author.
