# Supplementary material for: Symptom Patterns, Recovery, and Impact of Long COVID: Findings From a Longitudinal Survey
Source: Open Forum Infect Dis. 2026 Feb 27;13(2):ofag040. doi: 10.1093/ofid/ofag040 (PMC12947157; doi:10.1093/ofid/ofag040)
Supplement: ofag040_Supplementary_Data [file ofag040_supplementary_data.docx]

Supplementary Box 1: Questions used in the survey to capture reported measures

1. **Have you lost your job or had to give up work due to feeling unwell with COVID-19?**
   - Not applicable
   - No
   - No but I was furloughed
   - Yes – lost job
   - Yes – resigned/left job
2. **Has your COVID-19 illness resulted in loss of income?**
   - No
   - Yes
   - Not applicable
3. **What is your current employment status? Tick all that apply.**
   - Employed – full-time
   - Employed – part-time
   - Employed - phased return to work
   - Employed – working reduced hours
   - Employed – full-time and working reduced hours
   - Employed – part-time and working reduced hours
   - Self-employed or freelance without employees
   - Self-employed with employees
   - Unemployed and looking for work
   - Volunteer
   - Job training/apprenticeship
   - Student
   - At home and not looking for work (e.g. looking after home and/or family)
   - Unable to work
   - Made redundant/took early retirement
   - Retired
   - Other, please specify
4. **How would you describe your health currently? Tick all that apply**

- I am still experiencing Long Covid symptoms
- I feel far from recovery
- I feel stable but have lower level of health/activity
- I feel stable and close to baseline health/activity
- I feel potential for relapse
- Not sure because my symptoms come and go
- Yes**,** I consider myself fully recovered (feeling as healthy as I did before infection and able to function at the same level of activity)

**If yes – how long were you symptom-free before considering yourself completely recovered?**

- 2-4 weeks
- 1-2 months
- 2-3 months
- 3-6 months
- more than 6 months

**If yes – over what period did your Long Covid last?**

- 4 weeks/1 month
- 2-3 months
- 3-6 months
- 6-9 months
- 9-12 months
- 12-15 months
- more than 15 months

1. **What is/was the nature of your symptoms?**
   - Constant (experienced at least one symptom every day)
   - Fluctuating but never completely goes away compared to pre-Covid health
   - Relapsing and remitting – have symptom-free periods in between relapses
   - Constant at the start for around 2 weeks but fluctuating in intensity now and never completely goes away compared to pre-Covid health
   - Constant at the start for at least 4 weeks but fluctuating in intensity now and never completely goes away compared to pre-Covid health
   - Constant at the start for around 2 weeks but relapsing and remitting now with healthy periods in between
   - Constant at the start for at least 4 weeks but relapsing and remitting now with healthy periods in between
   - Other – please specify

Supplementary Table 1: Selected descriptive variables from the baseline data to assess difference in sample characteristics at follow-up by response

|  | Baseline (non-responders) | | Follow-up | |
| --- | --- | --- | --- | --- |
|  | n | % | n | % |
| n | 1057 |  | 1153 |  |
| Age | 45.1, SD 12.6 |  | 47.7, SD 10.6 |  |
| 18-30 | 94 | 9.2 | 63 | 5.5 |
| 31-45 | 441 | 43.2 | 415 | 36.0 |
| 46-59 | 391 | 38.3 | 519 | 45.1 |
| ≥60 | 95 | 9.3 | 155 | 13.5 |
| Gender |  |  |  |  |
| Male | 152 | 14.8 | 173 | 15.0 |
| Female | 869 | 84.8 | 965 | 83.8 |
| Other | 4 | 0.4 | 14 | 1.2 |
| Ethnicity |  |  |  |  |
| White | 942 | 92.1 | 1096 | 95.4 |
| Mixed/Multiple ethnic groups | 32 | 3.1 | 23 | 2.0 |
| Asian | 27 | 2.6 | 24 | 2.1 |
| Black/African/Caribbean | 16 | 1.6 | 4 | 0.4 |
| Other | 6 | 0.6 | 2 | 0.2 |
| Baseline health before COVID-19 infection |  |  |  |  |
| Poor | 14 | 1.4 | 10 | 0.9 |
| Fair | 92 | 9.0 | 103 | 8.9 |
| Good | 288 | 28.1 | 297 | 25.8 |
| Very good | 431 | 42.1 | 478 | 41.5 |
| Excellent | 199 | 19.4 | 265 | 23.0 |
| Duration of illness, months (mean ± SD) | 7.1 ± 2.0 |  | 7.2 ± 1.7 |  |
| Pattern of illness |  |  |  |  |
| Constant throughout | 72 | 7.0 | 91 | 8.9 |
| Gradually got worse | 124 | 12.1 | 119 | 11.6 |
| Gradually got better | 67 | 6.6 | 90 | 8.8 |
| Fluctuating | 576 | 56.4 | 686 | 67.1 |
| Relapsing/Comes and goes | 183 | 17.9 | 163 | 15.9 |
| Symptom frequency |  |  |  |  |
| Daily | 738 | 72.7 | 850 | 73.9 |
| >3 times a week | 162 | 16.0 | 193 | 16.8 |
| Once a week | 42 | 4.1 | 41 | 3.6 |
| Once a fortnight | 23 | 2.3 | 18 | 1.6 |
| Once a month | 8 | 0.8 | 16 | 1.4 |
| <Once a month | 14 | 1.4 | 3 | 0.3 |
| Daily and reduced over time | 10 | 1.0 | 9 | 0.8 |
| Episodic | 13 | 1.3 | 17 | 1.5 |
| Variable | 5 | 0.5 | 3 | 0.3 |
| Lost job or had/chose to stop work |  |  |  |  |
| No | 782 | 76.8 | 914 | 79.7 |
| No but was furloughed | 78 | 7.7 | 63 | 5.5 |
| Yes | 158 | 15.5 | 170 | 14.8 |
| Had time off sick |  |  |  |  |
| No | 286 | 27.9 | 307 | 26.6 |
| No but was furloughed | 62 | 6.1 | 45 | 3.9 |
| Yes | 677 | 66.1 | 801 | 69.5 |

Supplementary Figure 1: Plot showing response combinations for current health at follow-up

Alternate text: Graph showing data on responses to question on current health at time of follow-up.


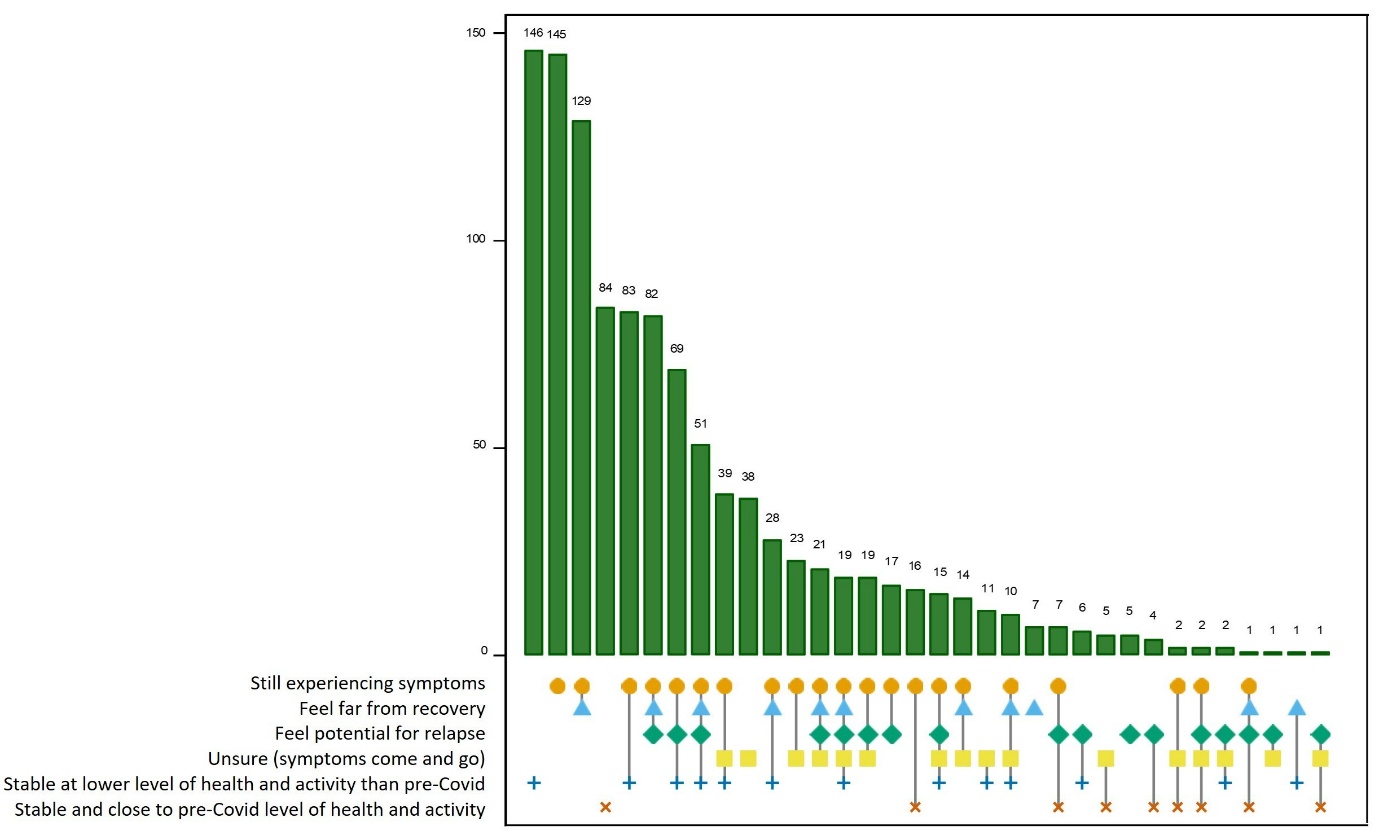


Supplementary Table 2: Long Covid symptoms reported at first onset, at time of baseline survey and at time of follow-up

|  | At onset/first two weeks of being symptomatic | | At time of baseline survey completion | | At time of follow-up | |
| --- | --- | --- | --- | --- | --- | --- |
|  | n | % | n | % | n | % |
| No symptoms – recovered or in long term remission | - | - | 15 | 1.3 | 66 | 6.0 |
| Fever | 597 | 51.8 | 90 | 7.8 | 81 | 7.4 |
| Cough | 681 | 59.1 | 285 | 24.8 | 239 | 21.7 |
| Altered or loss of sense of smell | 425 | 36.9 | 175 | 15.2 | 189 | 17.2 |
| Altered or loss of sense of taste | 418 | 36.3 | 155 | 13.5 | 166 | 15.1 |
| Abdominal pain | 255 | 22.1 | 207 | 18.0 | 211 | 19.2 |
| Diarrhoea | 394 | 34.2 | 196 | 17.0 | 201 | 18.3 |
| Loss of appetite | 422 | 36.6 | 148 | 12.9 | 124 | 11.3 |
| Nausea | 288 | 25.0 | 191 | 16.6 | 182 | 16.6 |
| Vomiting | 56 | 4.9 | 17 | 1.5 | 25 | 2.3 |
| Cognitive dysfunction |  |  |  |  |  |  |
| Brain fog | 363 | 31.5 | 726 | 63.1 | 685 | 62.3 |
| Confusion | 239 | 20.7 | 248 | 21.6 | 236 | 21.5 |
| Memory problems | 218 | 18.9 | 549 | 47.7 | 541 | 49.2 |
| Poor concentration | 328 | 28.5 | 558 | 48.5 | 562 | 51.1 |
| Difficulty processing information | - | - | - | - | 534 | 48.6 |
| Depression | 61 | 5.3 | 173 | 15.0 | 202 | 18.4 |
| Anxiety | - | - | 317 | 27.5 | 293 | 26.6 |
| Chest pain | 443 | 38.4 | 421 | 36.6 | 312 | 28.4 |
| Chest pressure | 606 | 52.6 | 463 | 40.2 | 381 | 34.6 |
| Chest tightness | 637 | 55.3 | 470 | 40.8 | 401 | 36.5 |
| Palpitations | 332 | 28.8 | 502 | 43.6 | - | - |
| Shortness of breath | 734 | 63.7 | 683 | 59.3 | 498 | 45.3 |
| Chills | 608 | 52.7 | 193 | 16.8 | 207 | 18.8 |
| Dizziness | 483 | 41.9 | 481 | 41.8 | 441 | 40.1 |
| Exhaustion | 883 | 76.6 | 878 | 76.3 | 746 | 67.8 |
| Headache | 753 | 65.3 | 560 | 48.7 | 496 | 45.1 |
| Eye symptoms | - | - | - | - | 213 | 19.4 |
| Hoarse voice | 312 | 27.1 | 222 | 19.3 | 219 | 19.9 |
| Nasal symptoms | 306 | 26.5 | 216 | 18.8 | 272 | 24.7 |
| Sore throat | 525 | 45.5 | 283 | 24.6 | 231 | 21.0 |
| Sneezing | 111 | 9.6 | 88 | 7.7 | 107 | 9.7 |
| Tinnitus | 141 | 12.2 | 308 | 26.8 | 373 | 33.9 |
| Joint pain | 393 | 34.1 | 451 | 39.2 | 507 | 46.1 |
| Leg pain | 263 | 22.8 | 300 | 26.1 | 342 | 31.1 |
| Muscle aches | 644 | 55.9 | 533 | 46.3 | 517 | 47.0 |
| Pins and needles | 175 | 15.2 | 311 | 27.0 | 319 | 29.0 |
| Skin rash | 132 | 11.5 | 143 | 12.4 | 160 | 14.6 |
| Sleep disturbance | 410 | 35.6 | 453 | 39.4 | 490 | 44.6 |
| Changes in menstruation | - | - | - | - | 167 | 15.2 |
| Post exertional symptom exacerbation | - | - | - | - | 687 | 62.5 |
| Hair loss | - | - | - | - | 192 | 17.5 |
| Acid reflux | - | - | - | - | 260 | 23.6 |
| Number of symptoms mean ± SD, median (interquartile range) | 12 ± 6  11 (7-16) | | 10 ± 6  10 (6-14) | | 10 ± 6  9 (5-14) | |
